# Low Dose of Paclitaxel Combined with XAV939 Attenuates Metastasis, Angiogenesis and Growth in Breast Cancer by Suppressing Wnt Signaling

**DOI:** 10.3390/cells8080892

**Published:** 2019-08-14

**Authors:** Dattatrya Shetti, Bao Zhang, Conghui Fan, Canlong Mo, Bae Hoon Lee, Kun Wei

**Affiliations:** 1 School of Biology and Biological Engineering, South China University of Technology, Guangzhou 510006, China; 2 Wenzhou Institute of Biomaterials and Engineering, University of CAS, Wenzhou 325011, China; 3 School of Biomedical Engineering, School of Ophthalmology & Optometry, Wenzhou Medical University, Wenzhou 325027, China

**Keywords:** breast cancer, XAV939, apoptosis, EMT, pristane, angiogenesis, combination treatment, Wnt signaling

## Abstract

Triple-negative breast cancer (TNBC) accounts for 15% of overall breast cancer. A lack of estrogen receptor (ER), progesterone receptor (PR), and human epidermal growth factor receptor 2 (HER2 receptor) makes TNBC more aggressive and metastatic. Wnt signaling is one of the important pathways in the cellular process; in TNBC it is aberrantly regulated, which leads to the progression and metastasis. In this study, we designed a therapeutic strategy using a combination of a low dose of paclitaxel and a Wnt signaling inhibitor (XAV939), and examined the effect of the paclitaxel-combined XAV939 treatment on diverse breast cancer lines including TNBC cell lines (MDA-MB-231, MDA-MB-468, and BT549) and ER+ve cell lines (MCF-7 and T-47D). The combination treatment of paclitaxel (20 nM) and XAV939 (10 µM) exerted a comparable therapeutic effect on MDA-MB-231, MDA-MB-468, BT549, MCF-7, and T-47D cell lines, relative to paclitaxel with a high dose (200 nM). The paclitaxel-combined XAV939 treatment induced apoptosis by suppressing Bcl-2 and by increasing the cleavage of caspases-3 and PARP. In addition, the in vivo results of the paclitaxel-combined XAV939 treatment in a mice model with the MDA-MB-231 xenograft further confirmed its therapeutic effect. Furthermore, the paclitaxel-combined XAV939 treatment reduced the expression of β-catenin, a key molecule in the Wnt pathway, which led to suppression of the expression of epithelial-mesenchymal transition (EMT) markers and angiogenic proteins both at mRNA and protein levels. The expression level of E-cadherin was raised, which potentially indicates the inhibition of EMT. Importantly, the breast tumor induced by pristane was significantly reduced by the paclitaxel-combined XAV939 treatment. Overall, the paclitaxel-combined XAV939 regimen was found to induce apoptosis and to inhibit Wnt signaling, resulting in the suppression of EMT and angiogenesis. For the first time, we report that our combination approach using a low dose of paclitaxel and XAV939 could be conducive to treating TNBC and an external carcinogen-induced breast cancer.

## 1. Introduction

Breast cancer is endocrine-related cancer with high incidence and mortality rates in women worldwide. Over the past decades, the incidence rate of breast cancer has increased continuously despite an advance in early detection techniques and an awareness of breast cancer. Based on the molecular subtype, four different types of breast cancer have been identified: (1) Luminal A (HR+/HER2−) (71%), (2) luminal B (HR+/HER2+) (12%), (3) HER2-enriched (HR-/HER2+) (5%), and (4) triple-negative (HR−/HER2−) (12%) [1]. Triple-negative breast cancer (TNBC) is responsible for 12%–15% of all breast cancer cases. The lack of estrogen receptor (ER), progesterone receptor (PR), and human epidermal growth factor receptor (HER2) in TNBC makes it highly aggressive and resistant to hormonal therapy [2,3]. The tendency of TNBC to metastasize to distinct organs is higher compared to other cancer phenotypes. Once cells get detached from the invasive front of TNBC, they migrate to distinct tissue sites through a capillary and proliferate in the new microenvironment [4]. TNBC is more sensitive to chemotherapy compared to other types of breast cancer. However, even after a series of therapies (a traditional radiotherapy, an adjuvant chemotherapy, a systematic therapy, and a targeted therapy), the peak risk of TNBC recurrence takes place within 3–5 years, ultimately leading to death in most of the cases [5,6]. Therefore, to ease the burden of TNBC treatment, an optimal therapeutic approach should be found.

Wnt signaling is an essential cellular process associated with cell fate (development, differentiation, and proliferation), but it is inappropriately regulated in multiple types of cancer [7,8]. Currently, the Wnt signaling pathway is characterized into β-catenin-dependent Wnt signaling (canonical pathway), β-catenin-independent Wnt signaling (non-canonical pathway), and the noncanonical Wnt/calcium pathway. All three pathways are triggered by a Wnt protein-ligand binding to a Frizzled family receptor, leading to the activation of downstream proteins. The main function of the canonical Wnt pathway is to stabilize β-catenin and enhance its nuclear translocation. Next, inside the nucleus, the transcription factor LEF/TCF (lymphoid enhancer-binding factor1/T-cell factor) is activated by active β-catenin, which in turn activates the target genes such as c-Myc, cyclin D, Axin2, and Nkd1 [9,10,11]. In the case of a lack of Wnt, β-catenin is degraded by the tumor suppressors such as adenomatous polyposis coli (APC), axis inhibitor (Axin), and glycogen synthase kinase 3 (GSK3), and a low level of β-catenin is maintained. 

In breast cancer, the Wnt signaling pathway is aberrantly regulated and both canonical and noncanonical pathways are involved in TNBC tumorigenesis [11,12]. The mutation in APC and β-catenin is rare in breast cancer, but TNBC tumorigenesis is thought to be promoted by an elevated level of β-catenin [13,14]. Mouse cancer model studies showed that accumulation of β-catenin in the nucleus was correlated with cell migration, colony formation, stem-like features, and chemoresistance in TNBC, thus suggesting that Wnt/ β-catenin activation in cells could lead to TNBC tumorigenesis [11]. The Wnt signaling destruction complex consists of Axin, the serine–threonine kinases casein kinase 1α/β (CK1), glycogen synthase kinase 3α/β (GSK3), and adenomatous polyposis coli (APC), which can play an important role in degrading β-catenin by phosphorylation [15]. Abnormal assembly of the destruction complex prevents the phosphorylation of β-catenin, resulting in the accumulation of β-catenin in the cytosol and the translocation of β-catenin to the nucleus [16,17]. The β-catenin destruction complex is highly regulated by the Axin concentration, and the degradation of β-catenin increases in a cell line with Axin overexpression, meaning that the Axin level is strictly controlled to maintain Wnt signaling. Axin levels are controlled by many processes; in fact, Wnt signaling itself regulates the Axin expression by promoting Axin degradation [18,19]. Thus, it is considered that an antagonist against Wnt signaling is an attractive tool for cancer treatment.

XAV939 (a small molecule inhibitor of Tankyrase) was found to successfully inhibit Tankyrase activity and stabilize Axin in MDA-MB-231 breast cancer cells. Treatment with XAV939 alone effectively attenuated the Wnt signaling in serum-deprived breast cancer cells. Further, XAV939 also blocked the cell migration and suppressed the cell growth in the serum-deprived medium. However, XAV939 alone was not so effective in treating TNBC [20]. 

Paclitaxel is a well-established drug for the treatment of breast cancer. Due to its side effects at a high dose, it is important to reduce the dose of paclitaxel while maintaining its anticancer effect. In our study, we aimed to examine a potential therapeutic effect of a low dose of paclitaxel combined with XAV939 on various breast cancer lines (MDA-MB-231, MDA-MB 468, BT549, MCF-7, and T-47D), MDA-MB-231-xenografted mice, and pristane-induced breast tumor. Here, we report that the combined treatment with XAV939 and a low dose of paclitaxel could treat TNBC effectively and synergistically. 

## 2. Material and Methods

### 2.1. Reagents

XAV939 was purchased from Selleckchem (Houston, TX, USA), paclitaxel and pristane were purchased from Aladdin (Shanghai, China). Dulbecco’s modified Eagle’s medium (DMEM) and Roswell Park Memorial Institute (RPMI) 1640 medium were purchased from Gibco (ThermoFisher, Gaisburg, MD, USA). Fetal bovine serum (FBS) and penicillin streptomycin were purchased from (P&S, Gibco). Dimethyl sulfoxide (DMSO) was purchased from MP Biomedicals (Solon, OH, USA), crystal violate was purchased from KeyGen BioTECH (Nanjing, China). MTT cell proliferation and cytotoxicity assay kit were purchased from Phygene Life Sciences (Fuzhou, China). Haematoxylin and eosin (H&E) staining kit was purchased from Servicebio, (Wuhan, China). Tunel assay kit was purchased from Roche (Indianapolis, IN, USA).

The primary antibody against β-catenin (D10A8) XP^®^, Axin1 (C7B12), Cleaved PARP (Asp214) (D64E10) XP^®^, Cleaved Caspase-3 (Asp175) (9664), and Bcl-2 (124) (15071) were purchased from Cell Signaling Technology (Danvers, MA, USA). Primary antibodies for IHC anti-MMP9 (GB12132), anti-vimentin (GB11192), anti-E-cadherin (GB11082), anti-Ki-67 (GB13030) and anti-β-catenin (GB12015) were purchased from Serveicebio (Woburn, MA, USA). Secondary antibodies against anti-mouse and anti-rabbit were purchased from (Beijing Zhongshang Jinqiao Biotechnology Co., Ltd. Beijing, China).

### 2.2. Cell Culture

Human breast cancer cell lines (MDA-MB-231, MDA-MB-468, BT549, MCF-7, and T-47D) were obtained from Procell (Wuhan, China). Cells were maintained in DMEM and RPMI 1640 supplemented with 10% FBS and 1% penicillin-streptomycin (100 U/mL penicillin and 100 mg/mL streptomycin) in an incubator with a 5% CO_2_ humidified atmosphere at 37 °C.

### 2.3. Cytotoxicity Assay

To assess the cytotoxicity effect of paclitaxel and/or XAV939 on MDA-MB-231, MDA-MB-468, BT549, MCF-7, and T-47D cells, the colorimetric MTT assay was performed as per the manufacturer’s instructions. Briefly, 8000 cells were seeded in a 96-well plate and treated with DMSO (3 µL/mL), different concentrations of paclitaxel (20 nM, 40 nM, and 200 nM) and XAV939 (5 µM and 10 µM), and a combination of paclitaxel and XAV939 (20 nM + 5 µM, 20 nM + 10 µM, 40 nM + 5 µM, and 40 nM + 10 µM) for 24 h, 48 h, and 72 h. After incubation, 110 µL of the MTT solution was added and incubated for 4 h. The purple precipitate was dissolved by adding 100 µL isopropanol and then measured at 570 nm using an EnSpire Multimode Plate Reader (PerkinElmer, Waltham, MA, USA). 

### 2.4. Cell Cycle by Flow Cytometery

Briefly, MDA-MB-231 cells (1 × 10^6^) were seeded in 6-well plates and after 24 h cells were treated with DMSO (3 µL/mL), paclitaxel (20 nM and 200 nM), XAV939 (10 µM), and paclitaxel + XAV939 (20 nM + 10 µM) for 48 h. After trypsinization, cells were fixed with 70% ethanol and incubated at 4 °C for 30 min. Next, cells were washed and suspended in PBS containing 0.5 mg/mL ribonuclease A (Sigma-Aldrich, St. Louis, MO, USA) and incubated for 30 min at 37 °C. Cells were stained with 50 μg/mL propidium iodide (Sigma-Aldrich, St. Louis, MO, USA) and analyzed with BD Accuri C6 Plus (BD) at an excitation wavelength of 488 nm and an emission wavelength of 630 nm. The cell cycle distribution was quantified by using BD Accuri C6 Plus software.

### 2.5. Annexin V/Propidium Iodide Staining

Annexin V/propidium iodide staining for apoptosis was conducted using Annexin V-FITC Detection kit (DojinDo, Kumamoto, Japan) according to the manufacturer’s instructions. Briefly, MDA-MB-231 cells (1 × 10^6^) were seeded in a 6-well plate, and after 24 h cells were treated with DMSO (3 µL/mL), Paclitaxel (20 nM), XAV939 (10 µM), paclitaxel + XAV939 (20 nM + 10 µM), and paclitaxel (200 nM). After 48 h, cells were trypsinized, single-cell suspensions were prepared with 5 μL of annexin V-FITC and subsequently with a 5 μL PI solution in the dark, and cell suspensions were incubated for 15 min. Then, a 400 μL annexin V Binding solution was added to each cell suspension, and each cell solution was analyzed by flow cytometry. Necrotic, early, and late apoptosis cells were identified by BD Accuri C6 Plus software (Version 227, BD Biosciences, San Jose, CA, USA). For microscopic observation, cells were deposited on slides, air dried, and the cover slip was mounted with a drop of the antifluorescent quencher (Beyotime, Shanghai, China) and observed under a confocal microscope Nikon Ti-E-A1 (Tokyo, Japan). 

### 2.6. Wound Healing Assay

To study the wound migration assay, MDA-MB-231 cells were used. Briefly, MDA-MB-231 cells (5 × 10^5^) were seeded in 24-well plates and grown in a monolayer. At 100% confluence, the sterile tip was used to make a straight wound, and then cells were treated with DMSO (3 µL/mL), paclitaxel (20 nM), XAV939 (10µ M), paclitaxel + XAV939 (20 nM + 10 µM), and paclitaxel (200 nM). Wound photos were taken at 0 h and 12 h under a microscope (Olympus). The extent of migration was measured by calculating the area of the remaining wound using Image J software (ImageJ Free ware, NIH, Bethesda, MD, USA, http//rsb.nih.gov/ij/) and expressed in the form of bar graphs.

### 2.7. Transwell Assay

Invasive ability of MDA-MB-231 cells was studied using a Transwell cell culture chamber (Corning, NY, USA). Briefly, MDA-MB-231 cells were treated with DMSO (3 µL/mL), paclitaxel (20 nM), XAV939 (10 µM), paclitaxel + XAV939 (20 nM + 10 µM), and paclitaxel (200 nM) for 24 h. MDA-MB-231 cells (2.5 × 10^4^) were harvested and seeded on the upper part (8.0 μm pore polycarbonate membrane) of the Boyden chamber coated with Matrigel (Corning). The Matrigel coating was conducted at a serum-free DMEM: Matrigel ratio (V/V) of 8 to 1 at 37 °C for 2 h. At the lower chamber, a 900 μL complete medium containing 5% FBS was added as chemoattractant. After 24 h incubation, migrated cells on the lower surface of the transwell membrane were fixed with paraformaldehyde and stained with 1% crystal violate. The number of migrated cells was counted from each well and photographed at 20× (Olympus). The experiment was repeated three times.

### 2.8. Nuclear Staining

To study the nuclear morphology of MDA-MB-231 and MCF-7 cells, Hoechst staining was performed. MBA-MB-231 (1 × 10^5^ cells) and MCF-7 (1 × 10^5^ cells) were seeded separately on the cover slip in a 24-well plate and then each cell line was treated with paclitaxel (20 nM), XAV939 (10 µM), paclitaxel + XAV939 (20 nM + 10 µM), and paclitaxel (200 nM) for 72 h, respectively. The cells were fixed for 20 min with 4% PFA, washed with PBS, and incubated with 0.3% Triton-X 100 for 5 min. The cells were washed with PBS, and Hoechst 33258 (Beyotime, Shanghai, China) was added to cells for 30 min in the dark. The cover slip was mounted with a drop of the antifluorescent quencher (Beyotime, Shanghai, China). The stained nuclei were photographed under the LSM 710 Laser scanning confocal microscope at 40× (Carl Zeiss, Oberkochen, German).

### 2.9. Immunofluorescence Study

To study the cytoskeleton reorganization in a breast cancer cell line (MDA-MB-231), an immunofluorescence technique was used. MDA-MB-231 cells (1 × 10^5^) were seeded on cover slips in a 24-well plate. Cells were treated with paclitaxel (20 nM), XAV939 (10 µM), paclitaxel + XAV939 (20 nM + 10 µM) and paclitaxel 200 nM, respectively for 48 h. Then, the treated cells were fixed with 2% paraformaldehyde for 15 min at room temperature and washed with PBS twice. Later, the cells were washed with a quenching solution (0.1% glycine in PBS) twice, permeated with 0.1% Triton X-100 for 10 min, and blocked using a blocking solution (10% FBS in PBS) for 1 h at room temperature. Afterwards, the cells were treated overnight with a primary antibody against Tubulin (anti α-Tubulin, 1:100, 2125, CST) followed by a secondary antibody—Alexa Fluor 488 conjugated goat anti-rabbit IgG (1:50, ZF-0511, Zhongshan Goldenbridge Biotechnology, Corp). To study the actin cytoskeleton reorganization, also the cells were stained with FITC conjugated phalloidin (Fushen, Shanghai, China). Also, nuclei were stained with 4,6-diamidino-2 phenylindole DAPI, and the cover slip was mounted with a drop of antifluorescent quencher (Beyotime, Shanghai, China). Immunofluorescence images were captured under a confocal microscope (Nikon Ti-E-A, Tokyo, Japan). Digital images were optimized for image resolution (a final resolution of 300 dpi), brightness, and contrast using Adobe Photoshop 7.0 (Adobe Systems, San Jose, CA, USA). No alteration was made to the image, such as addition or removal of image details. 

### 2.10. Western Blotting Analysis

Two breast cancer cell lines, MDA-MB-231 and MCF-7 cells (1 × 10^6^ cells/each well in 6-well plates), were treated with DMSO (3 µL/mL), paclitaxel (20 nM), XAV939 (10 µM), paclitaxel + XAV939 (20 nM + 10 µM), and paclitaxel (200 nM) for 48 h. Then, cells were lysed, and their proteins were isolated by using 1 mL of RIPA (Solarbio, Beijing, China) plus 10 µL of PMSF. The protein concentration of each cell lysate was measured with a BCA Protein Assay Kit (Sangon Biotech, Shanghai, China). Western blot analysis was carried out by separating 40 µg of each cell lysate via sodium dodecyl sulfate polyacrylamide gel electrophoresis (SDS-PAGE) and by electro-transferring it onto a polyvinylidene difluoride (PVDF) membrane (240 mA, 4 h). The membranes were blocked with 5% nonfat milk, washed with TBST buffer, and then incubated with primary antibodies—β-catenin (D10A8), Axin1 (C7B12), cleaved PARP (Asp214), cleaved caspase-3 (Asp175), and Bcl-2 (124) (15071) (Cell Signaling Technology) at a dilution of 1:1000 in Primary Antibody Dilution Buffer (Beyotime. Shanghai, China) at 4 °C overnight. Next, membranes treated with primary antibodies were washed 3 times with TBST and incubated with an anti-rabbit HRP-linked antibody at a 1:5000 dilutions for 1 h at room temperature. β-Actin was used as an internal reference. The signals were visualized by Immobilon Western Chemiluminescent HRP Substrate (Millipore Corporation, Billerica, MA, USA) with the Amersham Imager 600 imagers (GE Healthcare Life Science, Pittsburgh, PA, USA).

### 2.11. Pristane-Induced Mammary Tumorigenesis

All mice experiments were performed according to the institutional guidelines, following a protocol approved by the South China University of Technology Experimental Animal Center, Guangzhou, China. Pristane (200 µL, Aladdin Industrial, Shanghai, China) was injected in the left inguinal mammary fat pad of 6-week-old C57 female mice twice a week for up to 4 weeks [21]. About 50% of the pristane-treated mice population were able to induce a tumor near the mammary fat pad. Once the tumor volume became around 200 mm^3^–300 mm^3^, mice were segregated into four groups (3 mice/each group), and then mice were treated with paclitaxel (10 mg/kg), XAV939 (10 mg/kg), and a combination of paclitaxel (10 mg/kg) + XAV939 (10 mg/kg) at pristane-induced tumor sites twice a week for 4 weeks. Mice body weight and tumor volume were measured every week using a weighing machine and Vernier caliper. The tumor volume was calculated via using the formula V = (W (2) × L)/2 where V is the volume of the tumor, W is width of the tumor, and L is the length of the tumor. At designated time points, mice were sacrificed and photographed using a camera Canon 750d (Tokyo, Japan) 

### 2.12. Tumor-Xenografted Mice Model

All mice experiments were performed according to the institutional guidelines, following a protocol approved by the South China University of Technology Experimental Animal Center, Guangzhou, China. MDA-MB-231 cells (2.5–5 × 10^6^) were suspended in PBS and mixed with Matrigel at a ratio of 1:1. 150 µL. Suspended cells were injected subcutaneously into 6-week-old female BALB/c nude mice. Once a tumor was formed, mice were randomly segregated into four different groups (three mice/each group). Then, mice were treated with paclitaxel (10 mg/kg), XAV939 (10 mg/kg), and a combination of paclitaxel (10 mg/kg) + XAV939 (10 mg/kg) via intraperitoneal injection twice a week for 4 weeks. Mice body weight and tumor volume were measured every week with a weighing machine and Vernier caliper. The tumor volume was calculated via using the formula V = (W (2) × L)/2 where V is the volume of the tumor, W is the width of the tumor, and L is the length of the tumor. At designated time points, mice were sacrificed, and the tumor tissue in each mouse was weighed and photographed using a camera (Canon750d). For Western blot and PCR analyses, tumor tissues were snap-frozen. In addition, some of the tumor tissues were stored in 4% PFA for the immunohistochemistry and H&E staining.

### 2.13. Quantitative Real-Time RT-PCR

Total tumor RNA was isolated from snap-frozen tumor samples using TRIzol reagent (Sangon Biotech, Shanghai, China) according to the manufacture’s instruction. Single-stranded RNA was reverse transcribed into complementary DNA (cDNA) using ReverTra Ace^®^ qPCR RT Master Mix (TOYOBO LIFE SCIENCE, Shanghai, China) in T100™ Thermal Cycler (BIO RAD, Hercules, CA, USA). The sample mixture was prepared with 1.5 μL of cDNA in 10 μL of ChamQ SYBR qPCR Master Mix (Vazyme Biotech, Nanjing, China), primers, and ddH_2_O for a final volume of 20 μL. The primers for specific genes, which were purchased from Sangon Biotech (Shanghai, China), are mentioned below (Table 1). The LightCycler^®^ 96 Real-Time PCR System (Roche, Switzerland) was used to perform qPCR under the standard thermal conditions: 95 °C for 30 s, 40 cycles of 95 °C for 10 s, and 60 °C for 30 s, followed by 95 °C for 10 s, 65 °C for 60 s, and 97 °C for 1 s. GAPDH was used as an endogenous housekeeping gene. The threshold cycle (CT) was calculated in accordance with the 2^−ΔΔCt^ method relative to the expression of GAPDH. Three independent experiments were performed for each reaction in triplicate. 

### 2.14. Haematoxylin and Eosin Stain (H&E Stain)

Hematoxylin eosin staining was performed on paraffin-embedded tumor, kidney, liver, lung, and spleen tissues of MDA-MB-231-xenografted mice. Sections with a thickness of 4 µm were prepared, deparaffinized in xylene (two times), and rehydrated in a graded series of alcohol (two times 100% alcohol and 75% alcohol) and distilled water. Later, sections were stained with a hematoxylin solution, rinsed in water, passed through a 70% ethanol solution containing 1% HCl, and rinsed again with tap water. Sections were stained with eosin for 5 min and rinsed with absolute alcohol and xylene for 5 min. The images were taken by Pannoramic 250 Flash III (3D HISTECH, The Digital Pathology Company, Budapest, Hungary).

### 2.15. TUNEL Assay

TUNEL assay was performed to see nuclear DNA fragmentation in apoptotic cells using in situ Cell Death Detection Kit (Roche), according to the manufacturer’s instruction. In brief, paraffin sections of MDA-MB-231-xenografted mice tumor samples were deparaffinized in xylene and rehydrated in a series of graded alcohol, and permeabilized with proteinase K for 25 min 37 °C. The tissue sections were then incubated with TUNEL reaction buffer in a 37 °C humidified chamber for 2 h, and the nucleus was counterstained with DAPI for 1 min at room temperature. Section slides were covered with a drop of the antifade mounting medium. Stained apoptotic cells were visualized by confocal microscopy (Nikon Ti-E-A1).

### 2.16. Immunohistochemistry

Immunohistochemistry (IHC) was performed on 4 µm paraffin-embedded tumor sections of MDA-MB-231-xenografted mice. Sections were deparaffinized with xylene and rehydrated in pure ethanol and gradient ethanol (85% and 75%) for 5 min per step. Then, section slides were placed in EDTS (pH9.0) and pressure-cooked with water. Slides were blocked using 3% H_2_O_2_ and washed with PBS. Primary antibodies against MMP9 (1:800 Serveicebio), E-cad (1:500 Serveicebio), vimentin (1:800 Serveicebio), Axin1 (1:50 CST), β-catenin (1:400 Serveicebio), and Ki-67(1:500 Serveicebio) were incubated for overnight at 4 °C. Next morning, slides were washed with PBS. Secondary antibody goat anti-rabbit IgG H&L (HRP) labelled with HRP was added and incubated for 50 min. A freshly prepared DAB chromogenic reagent was added to the dried slides as per the manufacture’s instruction, and the slides were counter-stained with hematoxylin. The slides were then rinsed with xylene for 5 min and mounted with a resin mounting medium. The images were taken by Pannoramic 250 Flash III (3D HISTECH, The Digital Pathology Company, Budapest, Hungary).

### 2.17. Statistical Analysis

All the experimental data are expressed as mean ± SD (standard deviation, n = 3). Statistical analysis was performed by one-way ANOVA by using GraphPad Prism 6.01 software. In all cases, *p* < 0.05 was considered statistically significant.

## 3. Results

### 3.1. Combination Treatment with Paclitaxel and XAV939 Inhibited the Viability of Various Breast Cancer Cells and Promotes Their Apoptosis

**Figure 1:**
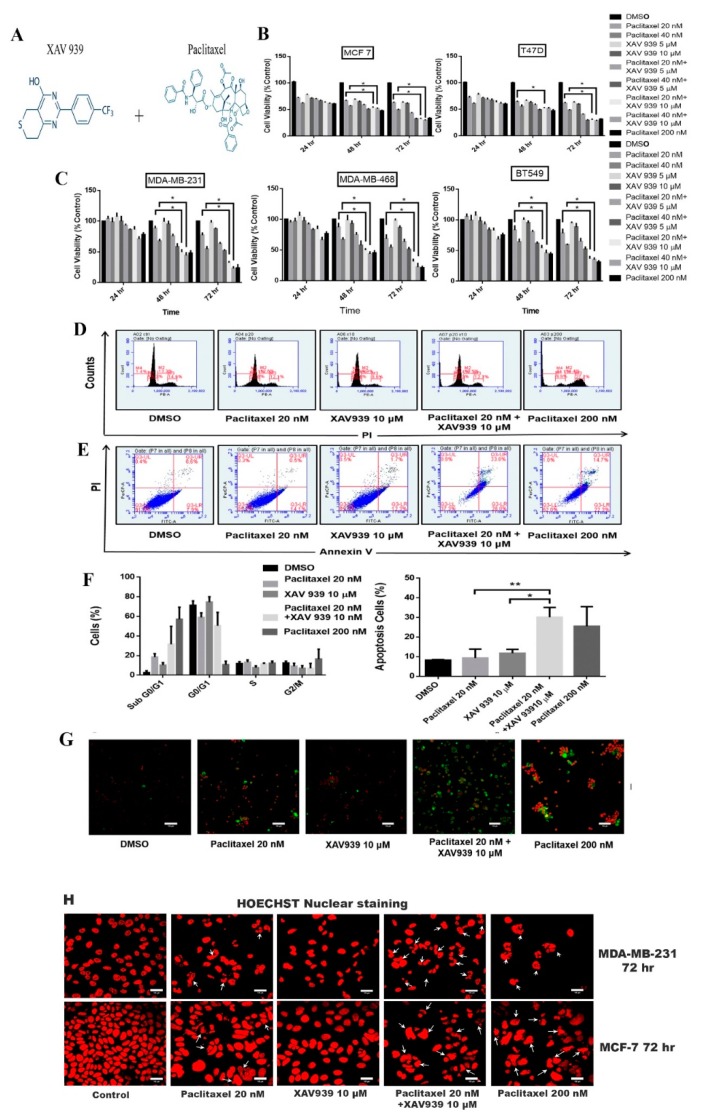
Combination treatment with paclitaxel (20 nM) and XAV939 (10 μM) induced comparable cytotoxicity in triple-negative breast cancer TNBC and estrogen receptor (ER)+ breast cancer cell lines, relative to paclitaxel with a high dose (200 nM). (**A**) The chemical structure of XAV939 and paclitaxel. (**B**,**C**) Cell viability of MDA-MB-231, MDA-MB-468, BT549, MCF-7, and T-47D cells after various treatments for 24 h, 48 h, and 72 h. (**D**) Cell cycle analysis of MDA-MB-231 treated with paclitaxel and/or XAV939 for 48 h. (**E**) MDA-MB-231 cells were treated with paclitaxel and/or XAV939 for 48 h and stained with annexin V-FITC and propidium iodide and then analyzed by flow cytometry. (**F**) The cell percentage of each cell cycle phase and the percentage of apoptotic cells in each treatment were analyzed statistically and presented in the form of bar graphs in three independent experiments. (**G**) Immunofluorescence images of apoptotic cells by annexin V-FITC and propidium iodide in MDA-MB-231 cells after various treatments for 48 h. (**H**) Nuclear fragmentation assay via Hoechst staining for MDA-MB-231 and MCF-7 cells after 72 h of various treatments. White arrows indicate the chromatin condensation and nuclear fragmentation. Results are expressed as mean ± SD, n = 3. *: *p* < 0.05; **: *p* < 0.001 in comparison to each single treatment with either paclitaxel or XAV939.

As seen in Figure S1 and Figure 1A, MDA-MB-231 cells were treated with paclitaxel (10 nM–100 nM) and XAV939 (0.3 µM–40 µM) separately at different doses for 48 h and 72 h. The cell viability of MDA-MB-231 was affected by Paclitaxel/XAV939 in a dose- and time-dependent manner. However, the cytotoxic effect of Paclitaxel was more evident than that of XAV939. Paclitaxel exhibited low cell viability (below 50%) for 72 h at a concentration above 30 nM whereas XAV939 showed relatively high cell viability (above 80%) across the treated concentrations. To confirm the cytotoxic effect of a combination of paclitaxel and XAV939 on TNBC cell lines (MDA-MB-231, MDA-MB-468, BT549) and ER+ve cells (MCF-7, T-47D), each cancer cell line was treated with paclitaxel (20 nM, 40 nM, and 200 nM), XAV939 (5 µM and 10 µM), and paclitaxel + XAV939 (20 nM + 5 µM, 20 nM + 10 µM, 40 nM + 5µM, and 40 nM + 10 µM), for 24 h, 48 h, and 72 h. Their cytotoxicity effect was measured by MTT assay. The combination treatment with paclitaxel + XAV939 at the concentrations of 20 nM + 10 µM and 40 nM + 10 µM caused more toxicity in a time-dependent manner compared to each single treatment with either paclitaxel or XAV939, as presented in Figure 1B,C. Interestingly, the cytotoxicity effect of the combination treatment employing a low dose of paclitaxel (20 nM) and XAV939 (10 µM) was almost equal to that of paclitaxel with a high dose (200 nM). Therefore, the combination treatment employing a low dose of paclitaxel (20 nM) and XAV939 (10 µM) were further investigated in the following experiments. In the cell cycle analysis of MDA-MB-231 cells, the combination treatment with paclitaxel and XAV939 increased the sub G0/G1 phase percentage in comparison to the single treatment with either paclitaxel (20 nM) or XAV939 (10 µM) as seen in Figure 1D. It was speculated that the combination treatment with paclitaxel + XAV939 (20 nM + 10 µM) might induce apoptosis or necrosis of MDA-MB-231 cells.

To confirm the apoptosis in sub G0/G1, we performed annexin V-FITC and PI assay. The percentage of cell apoptosis of the combination treatment with paclitaxel and XAV939 was much higher than of each single treatment with either paclitaxel (*p* < 0.001) or XAV939 (*p* < 0.05). On the other hand, the apoptotic percentage of the combination treatment was nearly equal to that of the treatment with a high dose of paclitaxel (200 nM), as seen in Figure 1E,F. The confocal images clearly depicted the significant increase in the florescence of annexin V-FITC (green) and PI (red) of the combination treatment with paclitaxel and XAV939, whereas the paclitaxel treatment with a high dose (200 nM) displayed the highest florescence of PI (red), as seen in Figure 1G. Figure 1H shows the nuclear morphology of apoptotic cells stained by Hoechst. The combined treatment with paclitaxel and XAV939 displayed the increased amount of the nuclear fragmentation and cell blebbing in MDA-MB-231 and MCF-7 cells, as compared to each single treatment with either paclitaxel or XAV939. c

### 3.2. Combination Treatment with Paclitaxel and XAV939 Inhibited Cell Migration and Invasion in Breast Cancer Cells

**Figure 2:**
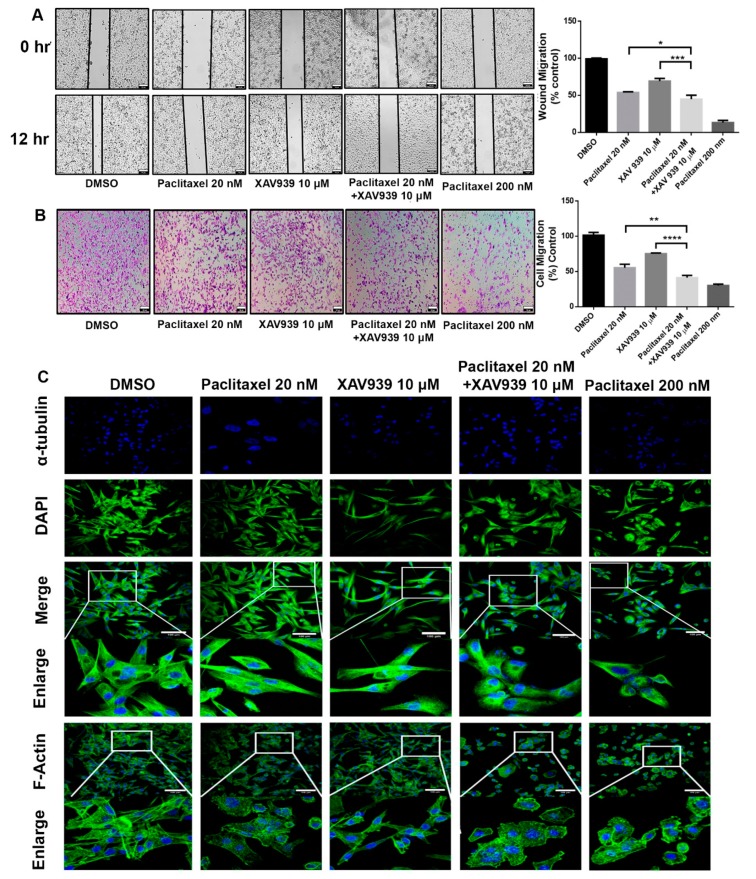
Combination treatment with paclitaxel (20 nM) and XAV939 (10 μM) inhibited the migration and invasion of MDA-MB-231 cells. (**A**) Cell scratch wound assay of MDA-MB-231 treated with paclitaxel and/or XAV939 for 12 h. Cells were photographed at a magnification of 4× (t = 0 h, t = 12 h) and analyzed by the image-j software. (**B**) Invasion assay of MDA-MB-231 cells treated with paclitaxel and/or XAV939 for 24 h. Migrated cells were stained with crystal violet and photographed at five different areas at a magnification of 4×. Colored cells were counted and analyzed statistically. (**C**) The cell integrity of MDA-MB-231 cells treated with paclitaxel and/or XAV939 for 48 h was examined via staining α-tubulin with anti-α-tubulin antibody. The actin disorganization in drug-treated MDA-MB-231 cells was examined using phalloidin (FITC) staining and observed under a confocal microscope. Data are presented as mean ± SD n = 3. *: *p* < 0.05; **: *p* < 0.001; ***: *p* < 0.0008, ****: *p* < 0.0001 in comparison to single treatment with either paclitaxel or XAV939. Scale bar = 100 μm.

Regarding the cytotoxicity effect of the combination treatment, the scratch wound assay and invasion assay were conducted to observe the cell migration of MDA-MB-231 in the presence of paclitaxel (20 nM) and/or XAV939 (10 µM). The combination treatment with paclitaxel and XAV939 showed more inhibited cell migration compared to each single treatment with either paclitaxel (*p* < 0.05) or XAV939 (*p* < 0.0008) at 12 h, as seen in Figure 2A. Similar trends were observed in the transwell invasion assay, as seen in Figure 2B. The number of invasive cells significantly decreased in the combination treatment with paclitaxel and XAV939 after 24 h, compared to each single treatment with either paclitaxel (*p* < 0.001) or XAV939 (*p* < 0.0001). Besides, the combination treatment with paclitaxel and XAV939 and paclitaxel with a high dose (200 nM) showed a similar number of migrated cells. Overall, the combination of paclitaxel (20 nM) + XAV939(10 µM) effectively inhibited cell migration and invasion in MDA-MB-231. To assess the cell integrity of cells after the treatments with paclitaxel and/or XAV939, cells were stained with the anti-α-tubulin antibody and with phalloidin (FITC). The combination treatment with paclitaxel and XAV939 as well as a high dose of paclitaxel (200 nM) damaged the cytoskeleton and destroyed cell integrity as displayed in Figure 2C. 

### 3.3. Combination Treatment with Paclitaxel and XAV939 Suppressed Wnt Signaling and Modulated Anti- and Proapoptotic Protein Expression in Breast Cancer Cells

**Figure 3:**
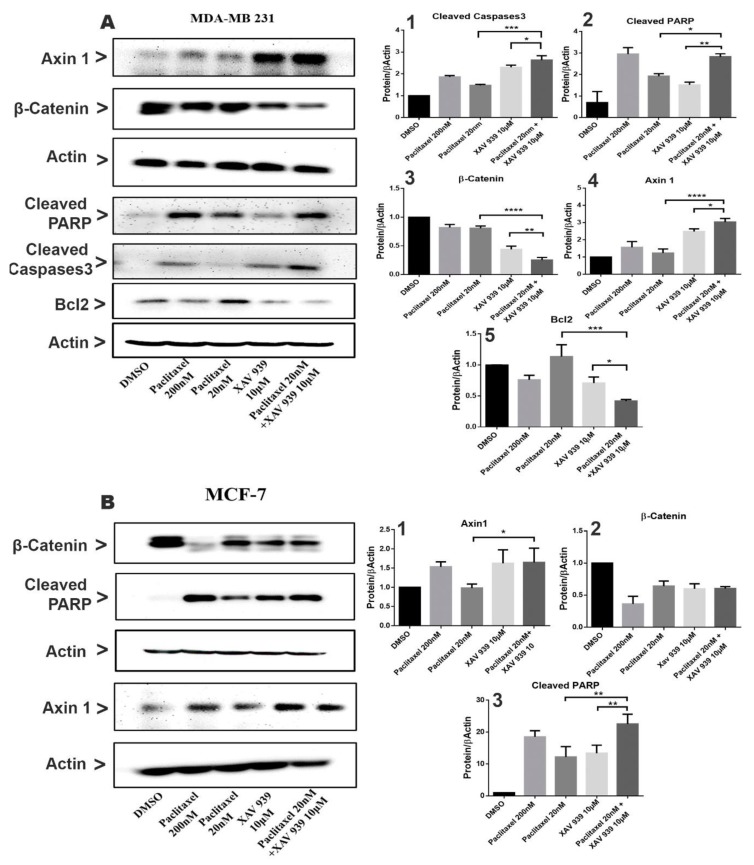
Combination treatment suppressed the Wnt signaling pathway and induced apoptosis in MDA-MB-231 and MCF-7 by the caspase-dependent pathway. (**A**) MDA-MB-231 cells were treated with paclitaxel and/or XAV939 for 48 h, and protein expression levels of β-catenin, Axin1, cleaved PARP, Bcl2, and cleaved caspases3 were analyzed using the Western blot. (**B**) The expression levels of β-catenin, Axin1, and cleaved PARP were analyzed in MCF-7 after treatment with paclitaxel and/or XAV939 for 48 h. All bar graphs represent the protein expression levels, which were quantified by densitometry from three different experiments and normalized to the expression level of actin. Results are expressed as mean ± SD (n = 3). *: *p* < 0.05; **: *p* < 0.01; ***: *p* < 0.005; ****: *p* < 0.001 in comparison to each single treatment with paclitaxel or XAV939.

To investigate the effect of paclitaxel (20 nM) and XAV939 (10 µM) combined or alone on Wnt signaling and apoptotic protein expression, the Western blot analysis was performed on MDA-MB-231 and MCF-7 cells as presented in Figure 3A,B. In MDA-MB-231 cells and MCF-7 cells, the single treatment of XAV939 (10 µM) had a relatively greater change in the expression of β-catenin and Axin1 in comparison to the control (DMSO, 3 µL/mL), whereas the single treatment with paclitaxel (20 nM) exhibited a smaller change in the expression of β-catenin and Axin1 in comparison to the control (DMSO).

In contrast, the combined treatment significantly decreased the β-catenin expression both in MDA-MB-231 and in MCF-7, whereas the expression of Axin1 in the combination treatment was noticeably increased in MDA-MB-231 (Figure 3(A3,A4,B1,B3)). The results indicate that the combination treatment can effectively inhibit the Wnt pathway in MDA-MB-231. In addition, the expression of apoptotic-related proteins such as cleaved Caspases3 and cleaved PARP in MDA-MB-231 and MCF-7 was almost nil in the control (DMSO), whereas each single treatment with paclitaxel or XAV939 increased their expression both in MDA-MB-231 and MCF-7. However, as seen in Figure 3(A1,A2), the combination treatment with paclitaxel and XAV939 exhibited a more noticeable increase in the expression levels of cleaved PARP (*p* < 0.05 or *p* < 0.01) and cleaved Caspases3 (*p* < 0.005 or *p* < 0.05) in MDA-MB-231 compared to each single treatment with paclitaxel or XAV939, whereas the combination treatment with paclitaxel and XAV939 displayed a higher rise in the expression level of cleaved PARP (*p* < 0.01 or *p* < 0.01) in MCF-7 cells, compared to each single treatment with paclitaxel or XAV939. As for the expression of Bcl-2, the combination treatment with paclitaxel and XAV939 significantly downregulated the expression of Bcl-2 in in MDA-MB-231 in comparison with each single treatment with paclitaxel (*p* < 0.005) or XAV939 (*p* < 0.05), as seen in Figure 3(A5). These results suggest that each single treatment of paclitaxel (20 nM) or XAV939 (10 µM) partially affected the Wnt signaling pathway and apoptotic process in MDA-MB-231 and MCF-7, whereas the combination treatment was actively involved in inhibiting the Wnt signaling pathway and inducing apoptosis.

### 3.4. Combination Treatment with Paclitaxel and XAV939 Suppressed Breast Tumor Growth in MDA-MB-231-Xenografted Tumor

**Figure 4:**
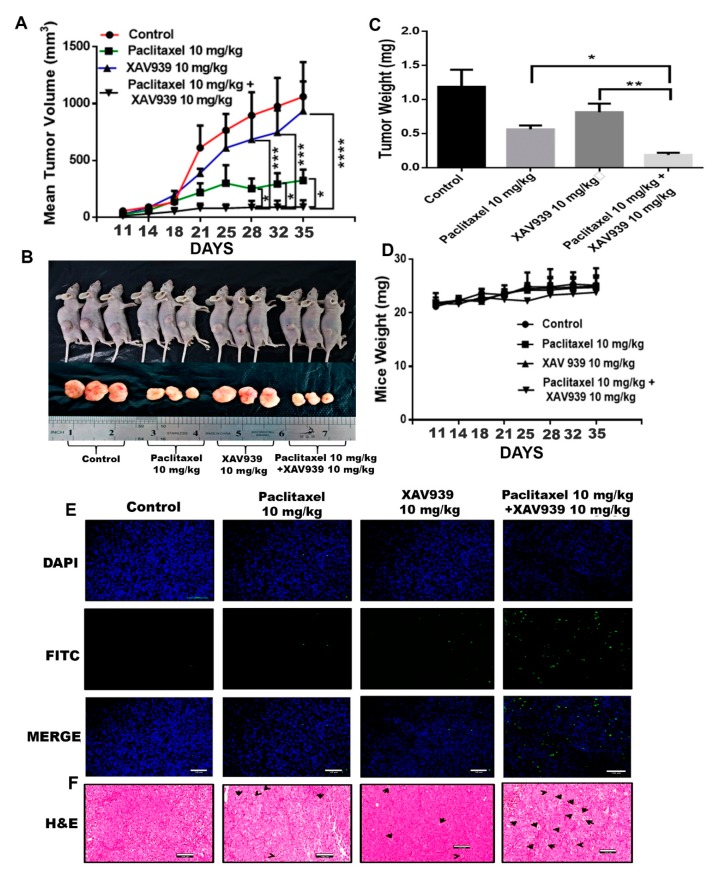
Combination treatment suppressed breast tumor growth by inducing apoptosis of tumor cells in an in vivo mouse model. MDA-MB-231-xenografted tumors were generated in BALB/c nude mice, and paclitaxel (10 mg/kg), XAV939 (10 mg/kg), and a combination of paclitaxel+XAV939 (10 mg/kg + 10 mg/kg) were injected intraperitoneally (i.p.) twice a week for 4 weeks. (**A**) Mouse tumor volume was monitored twice a week. Results are presented as mean ± SD (n = 3. *: *p* < 0.05, ***: *p* < 0.005, ****: *p* < 0.0003 compared to each single treatment with paclitaxel and XAV939). (**B**,**C**) At designated time points, mice were sacrificed, and tumors were isolated, photographed, weighed, and analyzed., Results are expressed as mean mass of tumors (mean ± SD, n = 3; *: *p* < 0.05, **: *p* < 0.001, compared to each single treatment with paclitaxel or XAV939). (**D**) The mouse weight was measured twice a week. (**E)** TUNEL assay was performed on the tumor section, and green dots represent the apoptotic cells. (**F**) H&E staining was performed on the tumor section, and black arrows indicate the apoptotic cells. Photographs were taken at a magnification of 20×. Scale bar = 100 μm.

The MDA-MB-231-xenografted mice tumor model was developed to study the impact of the paclitaxel and XAV939 combination treatment on breast tumor growth. After 10 days of MDA-MB-231 injection into mice, tumor-bearing mice were randomly distributed into four groups (three mice in each group). Treatment regimens, paclitaxel (10 mg/kg), XAV939 (10 mg/kg), and a combination of paclitaxel (10 mg/kg + XAV939 10 mg/kg), were injected intraperitoneally every two days a week until 4 weeks. In the course of the experiment, tumor length and breadth were measured twice a week using Vernier caliper. The tumor volume of the combination treatment was significantly smaller, compared to the control and each single treatment with paclitaxel (*p* < 0.05) or XAV939 (*p* < 0.001) Figure 4A. After the completion of the experiment, mice were sacrificed, and tumor samples were removed, photo-graphed, and weighed Figure 4B. As seen in Figure 4C, the tumor weight in the combination treatment was significantly lighter compared to each single treatment with paclitaxel (*p* < 0.05) or XAV939 (*p* < 0.001), but there was little or no effect of each drug on the body weight of mice Figure 4D. Altogether, our in vivo results demonstrate that the combination treatment with paclitaxel+ XAV939 could efficiently suppress the breast tumor growth, compared to the control and each single treatment. To correlate in vitro data with in vivo data, H&E staining and TUNEL assay were performed. In the TUNEL results, Figure 4E, the noticeable nuclear fragmentation in the tumor section of the combination treatment sample was observed, indicating the substantial apoptosis in the combination treatment sample. The H&E staining results of the tumors show that the combination treatment sample exhibited less infiltration of tumor cells and higher apoptotic cells, compared to the other treatments (Figure 4F).

### 3.5. Combination Treatment with Paclitaxel and XAV939 Suppressed Wnt Signaling and Modulated Anti- and Proapoptotic Protein in MDA-MB-231-Xenografted Tumors

**Figure 5:**
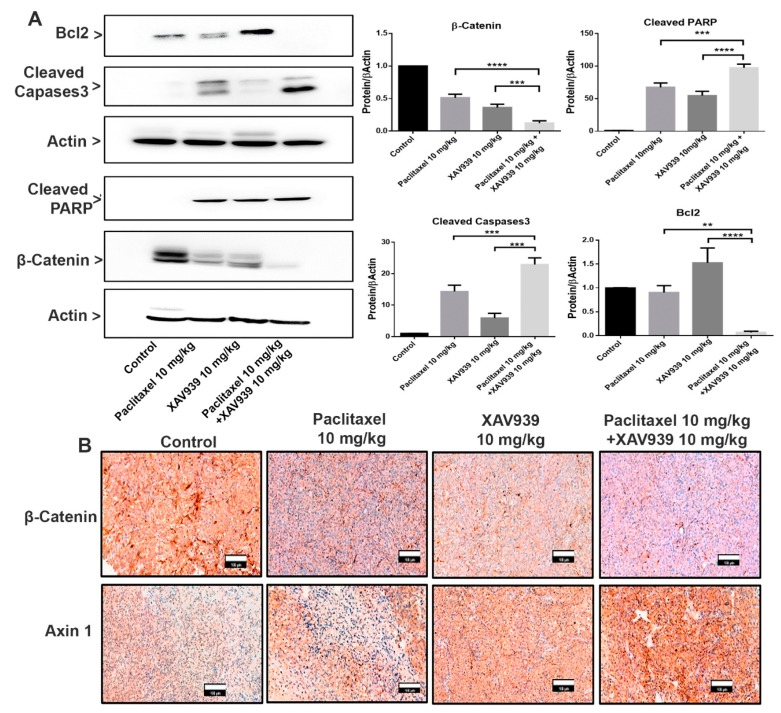
Combination treatment promoted the proapoptotic protein expression and suppressed the Wnt signaling pathway in an in vivo xenografted breast tumor model. (**A**) Tumor lysates were obtained, and the protein expression levels of β-catenin, cleaved PARP, Bcl2, and cleaved caspases3 were analyzed by the Western blot. Bar graphs represent the protein expression levels, which were normalized to the expression level of actin and quantified by densitometry from three different experiments (mean ± SD, n = 3; **: *p* < 0.01, ***: *p* < 0.005, and ****: *p* < 0.001, compared with each single treatment with paclitaxel or XAV939). (**B**) Tumor sections were subjected to immunohistochemical analysis of β-catenin and Axin1 using respective antibodies and were stained by diaminobenzidine (DAB, dark-brown staining). Photographs were taken at a magnification of 20×. Scale bar = 100 μm.

To examine the effect of paclitaxel (10 mg/kg), XAV939 (10 mg/kg), and a combination of paclitaxel (10 mg/kg) and XAV939 (10 mg/kg) on the Wnt signaling pathway and apoptotic protein expression, the Western blot and immunohistochemistry were performed on MDA-MB-231-xenografted mice tumor samples. As presented in Figure 5A, the combination treatment upregulated more significantly the protein expression of cleaved caspases 3 and cleaved PARP and suppressed the Bcl-2 expression level in tumor samples, compared to each single treatment with paclitaxel or XAV939. Moreover, the expression of Wnt signaling proteins were also measured as seen in Figure 5A,B. The combination treatment inhibited more significantly the β-Catenin expression level, compared to each single treatment with paclitaxel (*p* < 0.001) or XAV939 (*p* < 0.005), whereas the combination treatment with paclitaxel and XAV939 appeared to upregulate the expression of Axin1 in comparison with each single treatment with paclitaxel or XAV939 (Figure 5B). Overall, the results suggest that the combination treatment could suppress Wnt signaling and upregulate the apoptotic protein expression in the MDA-MB-231-xenografted tissue. 

### 3.6. Combination Treatment with Paclitaxel and XAV939 Suppressed the Epithelial-Mesenchymal Transition (EMT) Markers at Protein and mRNA Levels in MDA-MB-231-Xenografted Tumors

**Figure 6:**
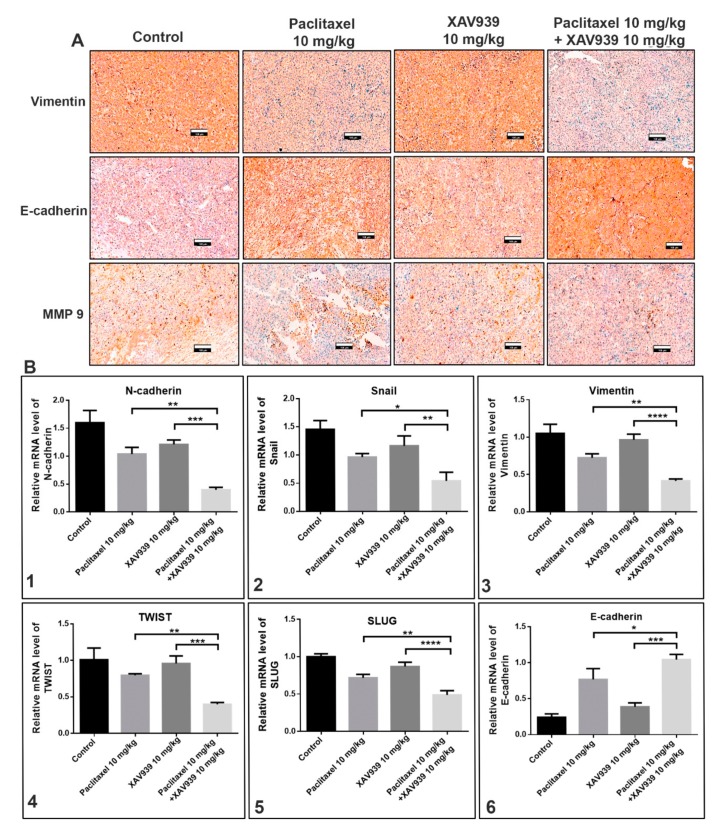
Combination treatment suppressed epithelial-mesenchymal transition (EMT) regulatory factors at protein and mRNA levels in MDA-MB-231-xenografted breast tumors. (**A**) Tumor sections were subjected to immunohistochemical analysis of vimentin, E-cadherin, and MMP9 using respective antibodies and stained by diaminobenzidine (DAB, dark-brown staining). Photographs were taken at a magnification of 20×. Scale bar = 100 μm. The tumor was lysed, and the mRNA expression levels of (**B1**) N-cadherin, (**B2**) Snail, (**B3**) vimentin, (**B4**) TWIST, (**B5**) SLUG, and (**B6**) E-cadherin were measured by RT-PCR. The relative expression of mRNA was normalized to the reference gene (GAPDH) and presented in a bar graph form. All data are expressed as mean ± SD (n = 3). *: *p* < 0.05, **: *p* < 0.005, ***: *p* < 0.001, and ****: *p* < 0.0001 when compared to each single treatment with paclitaxel or XAV939.

The effect of the combination treatment with paclitaxel and XAV939 on the expression of EMT markers in MDA-MB-231-xenografted mice tumor samples was assessed at protein and mRNA levels using immunohistochemistry and RT-PCR techniques. The protein expression of vimentin, MM9, E-cadherin in tumor samples was evaluated using immunohistochemistry, and mRNA levels of N-cadherin, Snail, vimentin, TWIST, SLUG, and E-cadherin were quantified by RT-PCR. As displayed in Figure 6A, the combination treatment with paclitaxel and XAV939 remarkably downregulated the expression (dark-brown staining) of vimentin and MMP9 (an EMT promoting protein), whereas the expression of E-cadherin (an EMT-inhibiting protein) was more significantly increased in the combination treatment with paclitaxel and XAV939 than in the control and each single treatment with paclitaxel or XAV939. 

To further assess whether the combination treatment could suppress the expression of EMT markers at mRNA level, we performed RT-PCR. As shown in the Figure 6B, the combination treatment effectively suppressed significantly the mRNA expression levels of N-cadherin (Figure 6(B1)), Snail (Figure 6(B2)), vimentin (Figure 6(B3)), TWIST (Figure 6(B4)), and SLUG (Figure 6(B5)), whereas the expression level of E-cadherin (Figure 6(B6)) was significantly increased in the combination treatment, compared to each single treatment with paclitaxel (*p* < 0.05) or XAV939 (*p* < 0.001).

### 3.7. Administration of the Combination Regimen of Paclitaxel and XAV939 Suppressed Cell Proliferation and Angiogenesis Markers at Protein and mRNA Levels in MDA-MB-231-Xenografted Tumor Models

**Figure 7:**
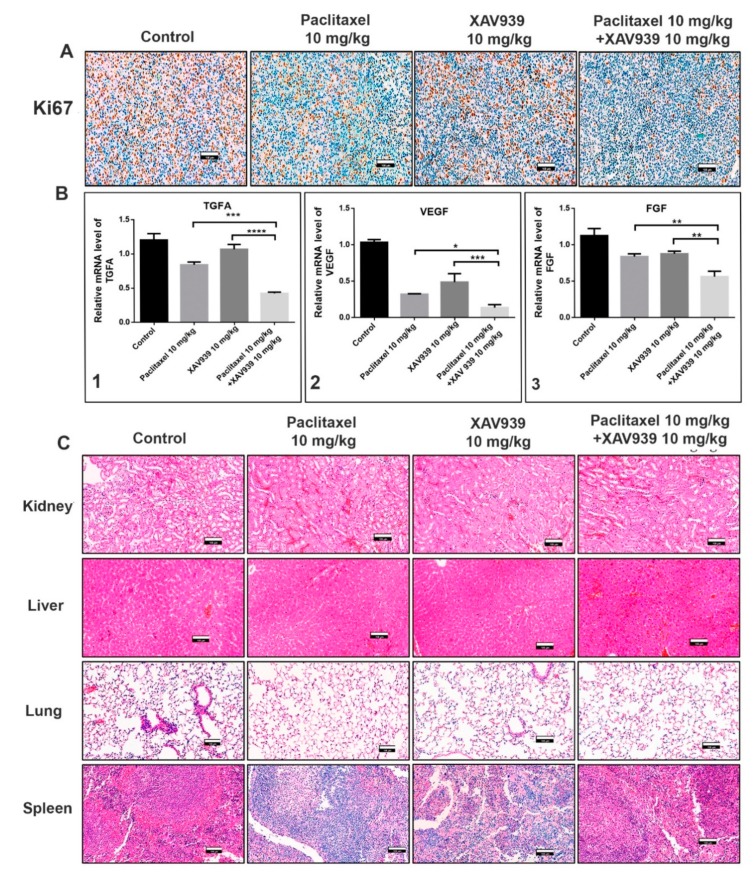
Combination regimen of paclitaxel and XAV939 suppressed cell proliferation and angiogenesis expression at protein and mRNA levels in MDA-MB-231-xenografted breast tumor models. (**A**) Tumor sections were subjected to immunohistochemistry (IHC) analysis for Ki-67 using anti-Ki-67 antibody and stained with DAB (a dark-brown color). The images represent the expression of Ki-67 in the control and the treatments with paclitaxel and/or XAV939. (**B**) The mRNA levels of (**B1**) TGFA, (**B2**) VEGF, and (**B3**) FGF were analyzed by real-time RT-PCR. The relative expression of mRNA was normalized to the reference gene (GAPDH) and presented in a bar graph form. All data are expressed as mean ± SD (n = 3). *: *p* < 0.03, **: *p* < 0.005, ***: *p* < 0.001, and ****: *p* < 0.0001, compared to each single treatment with either paclitaxel or XAV939. (**C**) H&E staining was performed to evaluate kidney, liver, lung, and spleen of the control and various drug-treated mice; photographs were taken at a 20× magnification. Scale bar = 100 μm.

To confirm whether the combination treatment can suppress the protein and mRNA expression related to cell proliferation and angiogenesis in MDA-MB-231-xenografted mice, immunohistochemistry and RT-PCR experiments were performed. Ki-67 is a nuclear protein and a well-accepted marker for cell proliferation. As seen in the immunohistochemistry results Figure 7A, the combination treatment with paclitaxel and XAV939 significantly reduced the Ki-67 expression (the dark-brown dots) as compared to the control and each single treatment with paclitaxel or XAV939. Next, we examined the mRNA level of angiogenic markers such as vascular endothelial growth factor (VEGF), transforming growth factor alpha (TGFA), and fibroblast growth factor (FGF) in the control and different treatments by RT-PCR. As presented in Figure 7B, the combination treatment with paclitaxel and XAV939 decreased the TGFA (Figure 7B1), VEGF (Figure 7B2), and FGF (Figure 7B3) expression at mRNA level as compared to each single treatment with paclitaxel (*p* < 0.05) or XAV939 (*p* < 0.0001).

Moreover, the safety of the combination regimen in mice was confirmed by the H&E staining of kidney, liver, lung, and spleen of the control and various drug-treated mice. Figure 7 shows that the combination regimen of paclitaxel and XAV939 had no adverse effect on the major organs of the breast tumor-xenografted mice, supporting its safety for breast cancer treatment. 

### 3.8. Combination Treatment with Paclitaxel and XAV939 Suppressed Pristane-Induced Breast Cancer in Female C57 Mice

**Figure 8:**
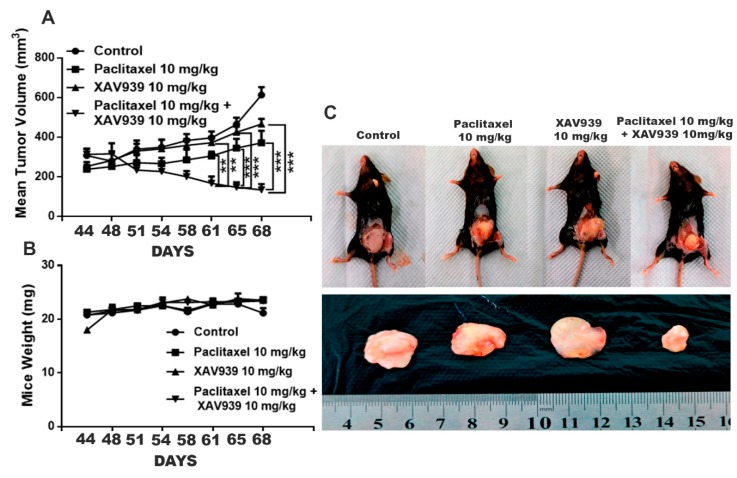
Combination treatment with paclitaxel and XAV939 suppressed the pristane-induced breast tumor in an in vivo mouse model. (**A**) The pristane-induced tumor was generated in C57 female mice, and paclitaxel (10 mg/kg), XAV939 (10 mg/kg), and a combination of paclitaxel + XAV939 (10 mg/kg + 10 mg/kg) were injected at a pristane-induced tumor site twice a week for 4 weeks. (**A**,**B**) Mouse tumor volume and body weight were measured every two weeks. Data were presented at mean ± SD (n = 3; **: *p* < 0.005 and ***: *p* < 0.0005, compared to each single treatment with paclitaxel or XAV939). (**C**) After 4 weeks, mice were sacrificed, and tumors were isolated and photographed.

It is well-known that an external chemical carcinogen can cause cancer in humans. To assess the impact of the combination treatment on an external carcinogen-induced breast cancer, pristane (a known carcinogen; 200 µL) was injected into the inguinal mammary fat pad of C57 female mice. After the confirmation of the tumor generation, paclitaxel of 10 mg/kg, XAV939 of 10 mg/kg, and a combination of paclitaxel 10 mg/kg + XAV939 10 mg/kg were administered twice a week at the pristane-induced tumor site. The combination regimen exhibited a significant decrease in the tumor volume growth, compared to each single regimen with either paclitaxel (after the 61-day treatment) or XAV939 (after the 51-day treatment), as seen in Figure 8A and Table S1. However, each drug treatment exhibited little or no effect on the mice body weight Figure 8B. At the end of the animal experiment, the mice were sacrificed and photographed; tumors were dissected and weighed Figure 8C. As seen in Figure 8C, the combination treatment with paclitaxel (10 mg/kg) + XAV939 (10 mg/kg) suppressed the growth of breast cancer induced by pristane more significantly than the other treatments.

## 4. Discussion

The Wnt signaling pathway is a crucial process in embryonic development and tissue homeostasis where cell surface receptors are initiated by a Wnt stimulus leading to β-catenin activation and transcription of Wnt-responsive genes. Based on the relation with β-catenin, Wnt signaling is classified into canonical (β-catenin-dependent) and noncanonical (β-catenin-independent) pathways. Dysregulation of Wnt signaling is a hallmark in many types of cancer, and breast cancer is no exception [7]. Studies have shown that canonical and noncanonical pathways are highly expressed in TNBC tumorigenesis and metastasis [11,12]. Moreover, in TNBC patients, the Wnt/β-catenin pathway is highly dysregulated, which leads to lung and brain secondary metastasis [22]. Over the past decades, inhibition of Wnt signaling was a universal strategy for treating cancer [23]. Recently, XAV939, a small molecule inhibitor of Wnt signaling has been identified [24]. XAV939 was found to selectively promote β-catenin degradation by stabilizing Axin1 and to suppress the Wnt signaling pathway. However, it has been reported that XAV939 alone could not efficiently inhibit metastasis and cell growth in the breast cancer cell line [24]. Therefore, a new strategy should be applied for treating TNBC. 

In this study, we tested a well-known cancer drug (paclitaxel) at a low toxic dose in combination with XAV939 for treating breast cancer. MTT results indicate that the combination treatment with paclitaxel and XAV939 is more effective in inhibiting cell proliferation of TNBC cell lines (MDA-MB-231, MDA-MB-468, and BT549) and ER+ve cell lines (MCF-7 and T-47D) compared to the single treatment with either paclitaxel or XAV939. The therapeutic effect of the combination regimen with a low dose of paclitaxel (20 nM) plus XAV939 (10 µM) was comparable to that of paclitaxel with a high dose (200 nM), confirming that the combination treatment is synergistically effective in inhibiting the growth of various breast cancer cells. The combination treatment with paclitaxel and XAV939 suppressed the proliferation of MDA-MB-231 more significantly than that of MCF-7. MDA-MB-231 cells are triple negative and highly aggressive, and their Wnt signaling is aberrantly regulated. It is important to find a strategy to tackle the growth and metastasis of MDA-MB-231 [25,26]. Cell cycle arrest is a major requirement of any cancer drug; most of the cancer drugs available on the market can induce cell cycle arrest in various phases of the cell cycle in cancer cells. Paclitaxel is known to bind to microtubules and to prevent their reorganization during cell division, subsequently resulting in cell cycle arrest. Also, the combination treatment with paclitaxel and XAV939 was found to interfere with the microfilament and microtubule structure in cancer cell lines. Moreover, in the treatment with paclitaxel and XAV939, cell apoptosis in MDA-MB-231 was induced at 48 h, and their DNA fragmentation was observed at 72 h, which suggests that the prolonged treatment with paclitaxel and XAV939 could trigger the apoptosis pathway like the treatment with a high dose of paclitaxel. Also, the combination treatment inhibited the canonical pathway in Wnt signaling by suppressing β-catenin expression and induced apoptosis in TNBC cells, in ER+ve cells, and in the tumor sample of the tumor-xenografted mice by suppressing Bcl-2 expression and by activating caspases3 that mediated PARP cleavage, ultimately leading to DNA condensation and fragmentation. In previous reports, during apoptosis, the expression of Bcl-2 was downregulated to facilitate the mitochondrial apoptotic pathway in which cytochrome C was released, and caspase 9 and caspase3 were activated, ultimately resulting in PARP cleavage [27,28]. Upregulation of PARP cleavage triggered an apoptotic signal and translocated the mitochondrial apoptosis-inducing factor (AIF) into the nucleus and provoked chromatin condensation and fragmentation [29]. The molecular mechanism by which the combination treatment can inhibit Wnt signaling and mediate apoptosis in MDA-MB-231 cells needs to be further studied. Nevertheless, the current study provides a basic platform for the further evaluation of the combination treatment against breast cancer.

EMT (epithelial to mesenchymal transition) is well associated with TNBC and plays a critical role in metastasis and progression of cancer [30]. In a recent study in TNBC patients, EMT markers have been established (vimentin, smooth muscle actin, osteonectin, and N-cadherin; loss of E-cadherin), and EMT inducers (ZEB1 and CD146) play an important role in EMT [31]. EMT is a complex process in which tumor cells secrete matrix metalloproteinases (MMPs). MMPs help in the degradation of the extracellular matrix (ECM) and allow tumors to lose their epithelial characteristics and to acquire mesenchymal phenotypes that are highly regulated by transcriptional factors such as Zeb1, Slug, Snail, TGF-β, and Twist [32,33]. Some of the transcriptional factors have been reported to play a role in angiogenesis [34]. Some studies have reported that the Wnt pathway plays an important role in promoting EMT and angiogenesis in cancer. Overexpression of Wnt3 ligand activated the Wnt/β-catenin pathway, whereas epidermal growth factor (EGF) secretion activated PI3K/Akt pathways, which accounted for β-catenin accumulation and mediated EMT and angiogenesis [35,36,37]. Since β-catenin is a major protein in the Wnt signaling pathway, targeting Wnt signaling can be a good strategy to control EMT and tumorigenesis. A previous report demonstrated that a small molecule inhibitor of Wnt signaling could inhibit cell migration and invasion in vitro [27]. Our results further disclose that a combination of a low dose of paclitaxel and a small molecule inhibitor (XAV939) can significantly suppress cancer cell migration and invasion in vitro and in vivo. In the TNBC-xenografted mice model, the combination treatment with paclitaxel and XAV939 efficiently downregulated the expression of β-catenin and stabilized Axin1, indicating the effective inhibition of the canonical pathway of Wnt signaling. Based upon the previous results, we speculate that our combination treatment with paclitaxel and XAV939 might inhibit the EMT pathway and angiogenesis in vivo. In fact, the combination treatment suppressed the expression of EMT regulating transcription factors (SNAIL, SLUG and Twist) at the mRNA level and inhibited the expression of pro-EMT markers (Vimentin, N-cadherin, and MMP 9) at the protein and mRNA levels. Besides, our combination treatment increased the E-cadherin level, which potentially corresponds to the inhibition of EMT. This inhibition was further correlated with the downregulation of proangiogenic molecules like Ki-67 in tumor samples, whereas the expression levels of VEGF, TGFA, and FGF were downregulated at mRNA level. Taken together, all these results suggest that the combination treatment with paclitaxel and XAV939 shows good potential in inhibiting various hallmarks of breast cancer.

In recent years, an external carcinogen is believed to be one of the major causes for cancer development. One study reported that an external carcinogen played a major role in altering the gene expression and activated oncogene genes, eventually inducing cancer progression [38]. Also, we studied the therapeutic effect of the combination treatment (paclitaxel and XAV939) on a carcinogen-induced breast tumor. The direct administration of the combination regimen significantly attenuated the growth and development of the pristane-induced breast cancer. 

Taken together, the single treatment with either a low dose of paclitaxel or XAV939 inhibited the Wnt pathway but could not significantly suppress TNBC breast cancer. Importantly, our strategy of combining a low dose of paclitaxel and XAV939 exhibited the strong antitumor activity in TNBC and inhibited EMT and angiogenesis by suppressing the canonical pathway of Wnt signaling in the MDA-MB-231-xenografted mice model. For the first time, we demonstrated that a combination of Tankyrase inhibitor i.e., XAV939 and a low dose of paclitaxel treatment could be a potential approach for treating TNBC cancer and carcinogen-induced breast cancer with minimum side effects.

## 5. Conclusions

In our study, we demonstrated that a low dose of paclitaxel combined with XAV939 (a Wnt inhibitor) enhanced the apoptotic effect in TNBC in comparison to each single treatment with paclitaxel or XAV939. The combination treatment with paclitaxel and XAV939 inhibited Wnt signaling, induced apoptosis, and suppressed EMT and angiogenesis in a TNBC tumor model. Additionally, the growth of breast cancer induced by pristane (a potent natural carcinogen) was suppressed by our combination treatment. All these findings propose that our strategy using a combination of a low dose of paclitaxel and XAV939 could have great potential in treating TNBC with little adverse effects. 

## Figures and Tables

**Table 1:** Primers used for reverse transcriptase polymerase chain reaction.

Name	Order	Primer Sequence
SNAI 2.	FORWARD	CTGTGACAAGGAATATGTGAGC
	REVERSE	CTAATGTGTCCTTGAAGCAACC
TGFA	FORWARD	GTGTCCCATTTTAATGACTGCC
	REVERSE	CACATGTGATGATAAGGACAGC
FGF1	FORWARD	CACAGACACCAAATGAGGAATG
	REVERSE	CATTCTTCTTGAGGCCAACAAA
CDH2	FORWARD	CGATAAGGATCAACCCCATACA
	REVERSE	TTCAAAGTCGATTGGTTTGACC
TWIST1	FORWARD	GTACATCGACTTCCTCTACCAG
	REVERSE	CATCCTCCAGACCGAGAAG
SNAI1	FORWARD	AATCCAGAGTTTACCTTCCAGC
	REVERSE	GAAGTAGAGGAGAAGGACGAAG
VIM	FORWARD	ATGTCCACCAGGTCCGTGT
	REVERSE	TTCTTGAACTCGGTGTTGATGG
CDH 1	FORWARD	AGTCACTGACACCAACGATAAT
	REVERSE	ATCGTTGTTCACTGGATTTGTG
VEGFA	FORWARD	ATCGAGTACATCTTCAAGCCAT
	REVERSE	GTGAGGTTTGATCCGCATAATC

## Supplementary Materials

The following are available online at https://www.mdpi.com/2073-4409/8/8/892/s1, Figure S1 Cell viability of MDA-MB-231 (8000 cells/each well) was assessed by MTT while MDA-MB-231 was treated with paclitaxel (PTX) and XAV939 at different doses for 48 h and 72 h. The cell viability of MDA-MB-231 was affected by paclitaxel and XAV939 in a dose and time dependent manner. However, the cytotoxic effect of paclitaxel was more evident than that of XAV939. The cell viability value of each drug was normalized to the cell viability value of DMSO (3 µL/mL). Table S1. C57 mice bearing the pristane-induced breast tumor were treated with paclitaxel (10 mg/kg), XAV939 (10 mg/kg), and a combination of paclitaxel+XAV939 (10 mg/kg + 10 mg/kg) twice a week for 4 weeks. (A) Tumor volume (mm^3^) and (B) mouse body weight (g) were monitored.

## References

[1] American Cancer Society Breast Cancer Facts & Figure 2017-2018. https://www.cancer.org/research/cancer-facts-statistics/breast-cancer-facts-figures.html.

[2] Carey L., Winer E., Viale G., Cameron D., Gianni L. (2010). Triple-negative breast cancer: Disease entity or title of convenience?. Nat. Rev. Clin. Oncol..

[3] Dalmau E., Armengol-Alonso A., Muñoz M., Seguí-Palmer M.Á. (2014). Current status of hormone therapy in patients with hormone receptor positive (HR+) advanced breast cancer. Breast.

[4] Fidler I.J. (2003). The pathogenesis of cancer metastasis: The “seed and soil” hypothesis revisited. Nat. Rev. Cancer.

[5] Malorni L., Shetty P.B., De Angelis C., Hilsenbeck S., Rimawi M.F., Elledge R., Osborne C.K., De Placido S., Arpino G. (2012). Clinical and biologic features of triple-negative breast cancers in a large cohort of patients with long-term follow-up. Breast Cancer Res. Treat.

[6] Guarneri V., Dieci M.V., Conte P. (2013). Relapsed Triple-Negative Breast Cancer: Challenges and Treatment Strategies. Drugs.

[7] Polakis P. (2012). Wnt signaling in cancer. Cold Spring Harb. Perspect. Biol..

[8] Clevers H., Nusse R. (2012). Wnt/β-catenin signaling and disease. Cell.

[9] Tetsu O., McCormick F. (1999). Beta-catenin regulates expression of cyclin D1 in colon carcinoma cells. Nature.

[10] Jho E., Zhang T., Domon C., Joo C.-K., Freund J.-N., Costantini F. (2002). Wnt/beta-catenin/Tcf signaling induces the transcription of Axin2, a negative regulator of the signaling pathway. Mol. Cell. Biol..

[11] Xu J., Prosperi J.R., Choudhury N., Olopade O.I., Goss K.H. (2015). β-Catenin Is Required for the Tumorigenic Behavior of Triple-Negative Breast Cancer Cells. PLoS ONE.

[12] Mohammed M.K., Shao C., Wang J., Wei Q., Wang X., Collier Z., Tang S., Liu H., Zhang F., Huang J. (2016). Wnt/β-catenin signaling plays an ever-expanding role in stem cell self-renewal, tumorigenesis and cancer chemoresistance. Genes Dis..

[13] Prosperi J.R., Goss K.H. (2010). A Wnt-ow of opportunity: Targeting the Wnt/beta-catenin pathway in breast cancer. Curr. Drug Targets.

[14] Turashvili G., Bouchal J., Burkadze G., Kolar Z. (2006). Wnt signaling pathway in mammary gland development and carcinogenesis. Pathobiology.

[15] Clevers H. (2006). Wnt/beta-catenin signaling in development and disease. Cell.

[16] Behrens J., Jerchow B.A., Würtele M., Grimm J., Asbrand C., Wirtz R., Kühl M., Wedlich D., Birchmeier W. (1998). Functional interaction of an axin homolog, conductin, with beta-catenin, APC, and GSK3beta. Science.

[17] Kishida M., Koyama S., Kishida S., Matsubara K., Nakashima S., Higano K., Takada R., Takada S., Kikuchi A. (1999). Axin prevents Wnt-3a-induced accumulation of beta-catenin. Oncogene.

[18] Leung J.Y., Kolligs F.T., Wu R., Zhai Y., Kuick R., Hanash S., Cho K.R., Fearon E.R. (2002). Activation of AXIN2 expression by beta-catenin-T cell factor. A feedback repressor pathway regulating Wnt signaling. J. Biol. Chem..

[19] Willert K., Shibamoto S., Nusse R. (1999). Wnt-induced dephosphorylation of axin releases beta-catenin from the axin complex. Genes Dev..

[20] Bao R., Christova T., Song S., Angers S., Yan X., Attisano L. (2012). Inhibition of tankyrases induces Axin stabilization and blocks Wnt signaling in breast cancer cells. PLoS ONE.

[21] Chakraborty G., Jain S., Patil T.V., Kundu G.C. (2008). Down-regulation of osteopontin attenuates breast tumour progression in vivo. J. Cell Mol. Med..

[22] Dey N., Barwick B.G., Moreno C.S., Ordanic-Kodani M., Chen Z., Oprea-Ilies G., Tang W., Catzavelos C., Kerstann K.F., Sledge G.W. (2013). Wnt signaling in triple negative breast cancer is associated with metastasis. BMC Cancer.

[23] Anastas J.N., Moon R.T. (2013). Wnt signaling pathways as therapeutic targets in cancer. Nat. Rev. Cancer.

[24] Huang S.-M.A., Mishina Y.M., Liu S., Cheung A., Stegmeier F., Michaud G.A., Charlat O., Wiellette E., Zhang Y., Wiessner S. (2009). Tankyrase inhibition stabilizes axin and antagonizes Wnt signaling. Nature.

[25] Khramtsov A.I., Khramtsova G.F., Tretiakova M., Huo D., Olopade O.I., Goss K.H. (2010). Wnt/beta-catenin pathway activation is enriched in basal-like breast cancers and predicts poor outcome. Am. J. Pathol..

[26] Geyer F.C., Lacroix-Triki M., Savage K., Arnedos M., Lambros M.B., MacKay A., Natrajan R., Reis-Filho J.S. (2011). β-Catenin pathway activation in breast cancer is associated with triple-negative phenotype but not with CTNNB1 mutation. Mod. Pathol..

[27] Will M., Qin A.C.R., Toy W., Yao Z., Rodrik-Outmezguine V., Schneider C., Huang X., Monian P., Jiang X., de Stanchina E. (2014). Rapid induction of apoptosis by PI3K inhibitors is dependent upon their transient inhibition of RAS-ERK signaling. Cancer Discov..

[28] Carmen J.C., Hardi L., Sinai A.P. (2006). Toxoplasma gondii inhibits ultraviolet light-induced apoptosis through multiple interactions with the mitochondrion-dependent programmed cell death pathway. Cell. Microbiol..

[29] Joza N., Susin S.A., Daugas E., Stanford W.L., Cho S.K., Li C.Y., Sasaki T., Elia A.J., Cheng H.Y., Ravagnan L. (2001). Essential role of the mitochondrial apoptosis-inducing factor in programmed cell death. Nature.

[30] May C.D., Sphyris N., Evans K.W., Werden S.J., Guo W., Mani S.A. (2011). Epithelial-mesenchymal transition and cancer stem cells: A dangerously dynamic duo in breast cancer progression. Breast Cancer Res..

[31] Jang M.H., Kim H.J., Kim E.J., Chung Y.R., Park S.Y. (2015). Expression of epithelial-mesenchymal transition-related markers in triple-negative breast cancer: ZEB1 as a potential biomarker for poor clinical outcome. Hum. Pathol..

[32] Huber M.A., Kraut N., Beug H. (2005). Molecular requirements for epithelial-mesenchymal transition during tumor progression. Curr. Opin. Cell Biol..

[33] De Craene B., Berx G. (2013). Regulatory networks defining EMT during cancer initiation and progression. Nat. Rev. Cancer.

[34] Singh S., Mak I.W.Y., Handa D., Ghert M. (2014). The Role of TWIST in Angiogenesis and Cell Migration in Giant Cell Tumor of Bone. Adv. Biol..

[35] Wu Y., Ginther C., Kim J., Mosher N., Chung S., Slamon D., Vadgama J.V. (2012). Expression of Wnt3 activates Wnt/β-catenin pathway and promotes EMT-like phenotype in trastuzumab-resistant HER2-overexpressing breast cancer cells. Mol. Cancer Res..

[36] Wang X., Wang S., Li X., Jin S., Xiong F., Wang X. (2017). The critical role of EGF-β-catenin signaling in the epithelial-mesenchymal transition in human glioblastoma. Onco Targets Ther..

[37] Parmalee N.L., Kitajewski J. (2008). Wnt signaling in angiogenesis. Curr. Drug. Targets.

[38] Cogliano V.J., Grosse Y., Baan R.A., Straif K., Secretan M.B., El Ghissassi F. (2005). Working Group for Volume 88 Meeting report: Summary of IARC monographs on formaldehyde, 2-butoxyethanol, and 1-tert-butoxy-2-propanol. Environ. Health Perspect..

