# Supplementary material for: Low Dose of Paclitaxel Combined with XAV939 Attenuates Metastasis, Angiogenesis and Growth in Breast Cancer by Suppressing Wnt Signaling
Source: Cells. 2019 Aug 14;8(8):892. doi: 10.3390/cells8080892 (PMC6721645; doi:10.3390/cells8080892)
Supplement: Supplementary file 1 [file cells-08-00892-s001.pdf]

**Title- Low dose of Paclitaxel Combined with XAV939 Attenuates Metastasis, Angiogenesis, and Growth in Breast Cancer by Suppressing Wnt Signaling**

**Dattatrya Shetti[1], Bao Zhang[1], Conghui Fan[1], Canlong Mo[1], Bae Hoon Lee[2,3]* and Kun Wei[1,2]***

[1] School of Biology and Biological Engineering, South China University of Technology, Guangzhou, Guangdong, 510006, China; dattakapilshetti@gmail.com (D.S.); Peoce@outlook.com (B.Z.); 1013298096@qq.com (C.F.); mocanlong9111@163.com (C.M.)

[2] Wenzhou Institute of Biomaterials and Engineering, University of CAS, Wenzhou, Zhejiang, 325011, China;

[3] School of Biomedical Engineering, School of Ophthalmology & Optometry, Wenzhou Medical University, Wenzhou 325027, Zhejiang, China

* Correspondence: bhlee@wibe.ac.cn; Tel.:+86-13676467727
weikun@scut.edu; Tel.: +86-13902848267

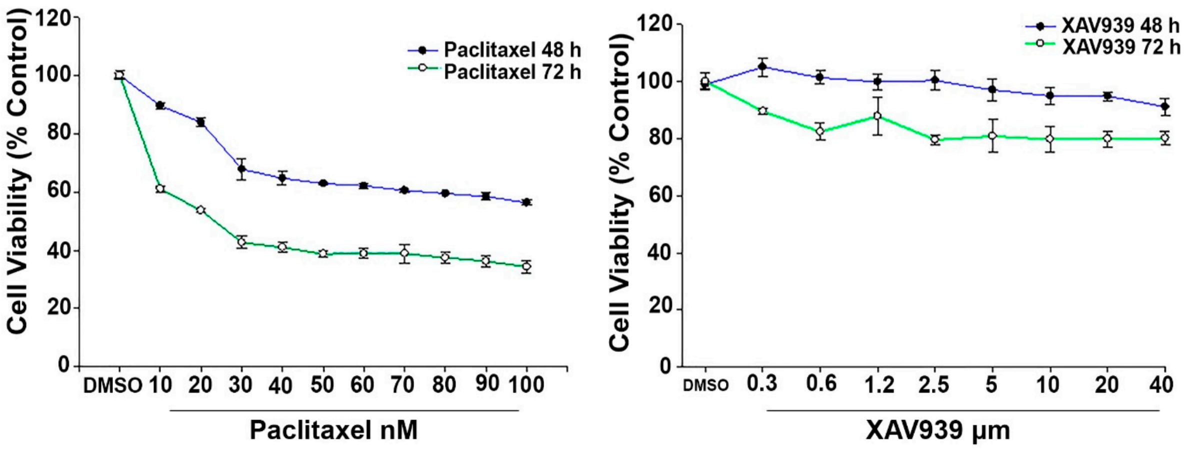


Figure S1. Cell viability of MDA-MB-231 (8000 cells/each well) was assessed by MTT while MDA-MB-231 was treated with Paclitaxel (PTX) and XAV939 at different doses for 48 h and 72 h. The cell viability of MDA-MB-231 was affected by Paclitaxel and XAV939 in a dose and time dependent manner. However, the cytotoxic effect of Paclitaxel was more evident than that of XAV939. The cell viability value of each drug was normalized to the cell viability value of DMSO (3 µL/mL).

Table S1. Tumor volume of C57 mice bearing the pristane-induced breast tumor and their body weight.

**A. Tumor Volume ($mm^3$)**

| Day | Control | | | Mean | STDEV | Paclitaxel 10mg/kg | | | Mean | STDEV | XAV939 10mg/kg | | | Mean | STDEV | Paclitaxel 10mg/kg + XAV939 10mg/kg | | | Mean | STDEV | P value: Between Paclitaxel 10mg/kg and Paclitaxel 10mg/kg + XAV939 10mg/kg | P value: Between XAV939 10mg/kg and Paclitaxel 10mg/kg + XAV939 10mg/kg |
|---|---|---|---|---|---|---|---|---|---|---|---|---|---|---|---|---|---|---|---|---|---|---|
| | 1 | 2 | 3 | | | 1 | 2 | 3 | | | 1 | 2 | 3 | | | 1 | 2 | 3 | | | | |
| 44 | 270.8 | 320.5 | 330.7 | 307.3 | 26.2 | 213.8 | 240.2 | 260.3 | 238.1 | 19.1 | 228.5 | 260.2 | 270.2 | 253.0 | 17.8 | 280.8 | 320.2 | 339.2 | 313.4 | 24.3 | 0.0262 | 0.0471 |
| 48 | 270.3 | 324.2 | 245.4 | 280.0 | 32.9 | 218.4 | 260.3 | 278.0 | 252.3 | 25.0 | 267.6 | 284.2 | 296.2 | 282.7 | 11.8 | 259.8 | 326.2 | 364.0 | 316.7 | 43.1 | 0.1414 | 0.3425 |
| 51 | 291.7 | 350.3 | 376.2 | 339.4 | 35.3 | 230.8 | 303.3 | 281.2 | 271.8 | 30.3 | 289.3 | 340.1 | 361.2 | 330.2 | 30.2 | 195.0 | 248.0 | 259.3 | 234.1 | 28.0 | 0.2666 | 0.0299 |
| 54 | 305.1 | 365.2 | 379.3 | 349.9 | 32.1 | 234.1 | 275.3 | 291.2 | 266.9 | 24.1 | 290.5 | 359.2 | 375.2 | 341.6 | 36.8 | 194.4 | 236.2 | 251.2 | 227.3 | 24.0 | 0.1749 | 0.0212 |
| 58 | 346.5 | 396.2 | 408.2 | 383.7 | 26.7 | 237.0 | 280.3 | 340.3 | 285.8 | 42.3 | 324.9 | 365.1 | 387.2 | 359.1 | 25.8 | 169.6 | 206.3 | 226.1 | 200.7 | 23.4 | 0.0676 | 0.0303 |
| 61 | 361.6 | 410.0 | 420.3 | 397.3 | 25.6 | 282.2 | 310.3 | 325.2 | 305.9 | 17.8 | 338.6 | 379.3 | 398.2 | 372.0 | 24.9 | 131.7 | 170.2 | 200.2 | 167.4 | 28.0 | 0.004 | 0.0051 |
| 65 | 427.3 | 478.2 | 489.3 | 465.0 | 27.0 | 302.2 | 336.0 | 396.2 | 344.8 | 38.9 | 395.1 | 430.2 | 450.1 | 425.1 | 22.8 | 117.5 | 159.3 | 165.2 | 147.3 | 21.2 | 0.0003 | 0.0002 |
| 68 | 567.9 | 630.3 | 640.3 | 612.9 | 32.0 | 324.5 | 350.2 | 440.2 | 371.6 | 49.6 | 438.7 | 470.3 | 491.0 | 466.7 | 21.5 | 100.5 | 154.3 | 148.2 | 134.3 | 24.1 | 0.0003 | 0.0001 |

**B. Mice body weight (g)**

| Day | Control | | | Mean | STDEV | Paclitaxel 10mg/kg | | | Mean | STDEV | XAV939 10mg/kg | | | Mean | STDEV | Paclitaxel 10mg/kg + XAV939 10mg/kg | | | Mean | STDEV | P value: Between Paclitaxel 10mg/kg and Paclitaxel 10mg/kg + XAV939 10mg/kg | P value: Between XAV939 10mg/kg and Paclitaxel 10mg/kg + XAV939 10mg/kg |
|---|---|---|---|---|---|---|---|---|---|---|---|---|---|---|---|---|---|---|---|---|---|---|
| | 1 | 2 | 3 | | | 1 | 2 | 3 | | | 1 | 2 | 3 | | | 1 | 2 | 3 | | | | |
| 44 | 20.4 | 21.0 | 20.9 | 20.8 | 0.3 | 21.1 | 21.0 | 21.9 | 21.3 | 0.4 | 17.7 | 17.9 | 18.4 | 18.0 | 0.3 | 20.9 | 21.5 | 19.8 | 20.7 | 0.7 | 0.3545 | 0.0072 |
| 48 | 21.0 | 21.6 | 20.9 | 21.2 | 0.3 | 21.9 | 22.6 | 20.8 | 21.8 | 0.7 | 21.7 | 22.5 | 21.1 | 21.8 | 0.6 | 21.6 | 22.1 | 21.1 | 21.6 | 0.4 | 0.7943 | 0.7546 |
| 51 | 21.1 | 21.6 | 22.3 | 21.7 | 0.5 | 22.4 | 22.9 | 22.1 | 22.5 | 0.3 | 21.8 | 22.4 | 21.1 | 21.8 | 0.5 | 22.0 | 21.3 | 22.1 | 21.8 | 0.4 | 0.124 | 0.9448 |
| 54 | 22.6 | 22.9 | 22.0 | 22.5 | 0.4 | 22.7 | 21.9 | 22.9 | 22.5 | 0.4 | 23.0 | 23.3 | 22.7 | 23.0 | 0.2 | 23.5 | 23.8 | 22.1 | 23.1 | 0.7 | 0.3553 | 0.8209 |
| 58 | 21.4 | 21.9 | 20.9 | 21.4 | 0.4 | 21.6 | 22.1 | 21.2 | 21.6 | 0.4 | 23.4 | 23.9 | 24.0 | 23.8 | 0.3 | 23.1 | 23.4 | 22.9 | 23.1 | 0.2 | 0.0073 | 0.0548 |
| 61 | 22.5 | 22.9 | 23.0 | 22.8 | 0.2 | 23.9 | 22.1 | 22.9 | 23.0 | 0.7 | 22.8 | 22.1 | 23.0 | 22.6 | 0.4 | 23.8 | 23.1 | 22.9 | 23.3 | 0.4 | 0.6367 | 0.1761 |
| 65 | 22.5 | 22.9 | 23.0 | 22.8 | 0.2 | 23.4 | 23.8 | 22.9 | 23.4 | 0.4 | 23.1 | 23.5 | 24.9 | 23.8 | 0.8 | 23.2 | 23.8 | 22.7 | 23.2 | 0.4 | 0.7651 | 0.3959 |
| 68 | 20.1 | 21.4 | 21.9 | 21.1 | 0.8 | 24.0 | 23.8 | 23.2 | 23.7 | 0.3 | 23.6 | 22.9 | 24.0 | 23.5 | 0.5 | 23.4 | 23.9 | 23.0 | 23.4 | 0.4 | 0.5462 | 0.8798 |

C57 mice bearing the pristane-induced breast tumor were treated with Paclitaxel (10 mg/kg), XAV939 (10 mg/kg), and a combination of Paclitaxel+XAV939 (10 mg/kg + 10 mg/kg) twice a week for 4 weeks. (A) Tumor volume ($mm^3$) and (B) mouse body weight (g) were monitored.
